# MicroRNA-325-3p Targets Human Epididymis Protein 4 to Relieve Right Ventricular Fibrosis in Rats with Pulmonary Arterial Hypertension

**DOI:** 10.1155/2022/4382999

**Published:** 2022-01-22

**Authors:** Yi Tang, Xiaowei Huo, Junyu Liu, Yijin Tang, Min Zhang, Wenlin Xie, Zhaofen Zheng, Jin He, Jiayan Lian

**Affiliations:** ^1^Department of Cardiology, Hunan Provincial People's Hospital, The First Affiliated Hospital of Hunan Normal University, Clinical Medicine Research Center of Heart Failure of Hunan Province, Hunan Normal University, Changsha, Hunan 410005, China; ^2^College of Pharmaceutical Science, Key Laboratory of Pharmaceutical Quality Control of Hebei Province, Hebei University, Baoding, Hebei 071002, China; ^3^Department of Cardiology, The First Affiliated Hospital of Hunan Normal University (Hunan Provincial People's Hospital), Hunan Normal University, Changsha, Hunan 410005, China; ^4^Department of Pathology, The Seventh Affiliated Hospital of Sun Yat-sen University, Shenzhen, Guangdong 518107, China

## Abstract

**Background:**

Pulmonary arterial hypertension (PAH) usually causes right ventricular dysfunction, which is closely related to cardiac fibrosis. But cardiac fibrosis mechanism remains unclear. Our purpose was to explore microRNA-325-3p (miR-325-3p)/human epididymis protein 4's (HE4) role in the occurrence and development of right ventricular fibrosis in PAH.

**Methods:**

The right ventricular fibrosis model of rats with PAH was constructed, and miR-325-3p was overexpressed to explore miR-325-3p's effect on myocardial fibrosis in rats with PAH. Pearson correlation coefficient examined the correlation between HE4 and miR-325-3p. We separated and identified the primary rat myocardial fibroblasts from the heart tissue. Then, the Ang II-treated myocardial fibroblast fibrosis model was constructed. miR-325-3p mimics and si-HE4 and oe-HE4 cell lines were constructed to investigate miR-325-3p and HE4 effects on myocardial cell fibrosis. Then, we added PI3K inhibitor LY294002 to study the effects of HE4 in cell fibrosis by the PI3K/AKT pathway. Starbase was used to predict miR-325-3p and HE4 binding sites. Dual-luciferase reporter assay verified whether miR-325-3p and HE4 were targeted.

**Results:**

Overexpression of miR-325-3p alleviated myocardial fibrosis in rats with PAH. Pearson correlation coefficient showed that HE4 was negatively correlated with miR-325-3p expression. Starbase predicted that miR-325-3p had binding sites with HE4. Dual-luciferase reporter assay demonstrated that miR-325-3p targeted HE4. Overexpression of miR-325-3p downregulated HE4 and inhibited myocardial fibroblast fibrosis. HE4 knockdown inhibited myocardial fibroblast fibrosis. HE4 promoted myocardial fibroblast fibrosis and activated the PI3K/AKT pathway. In addition, HE4 affected myocardial fibroblast fibrosis through the PI3K/AKT pathway.

**Conclusions:**

miR-325-3p could target HE4 to relieve right ventricular fibrosis in rats with PAH. This study could provide new targets and strategies for right ventricular fibrosis in PAH.

## 1. Introduction

Pulmonary hypertension (PH) is defined as mean pulmonary artery pressure (mPAP) > 20 mmHg at rest [1]. Pulmonary arterial hypertension (PAH) is a class of PH that seriously affects the pulmonary vascular system [2]. The PAH patients died of right heart failure despite the use of targeted drugs that interfere with the endothelin, nitric oxide, and prostacyclin pathways to the pulmonary vessels [3]. Right ventricular fibrosis is an important cause of right ventricular failure [4]. Right ventricular fibrosis in PAH patients is characterized by cardiac fibrogenesis caused by endothelium-mesenchymal transition and increased collagen production [5], but the mechanism of myocardial fibrosis remains unclear.

MicroRNA (miRNA) is a small noncoding RNA that plays a significant regulatory role in inhibiting cleavage or translational repression by targeting mRNA cutting or translation [6]. miRNAs are closely related to PAH development. miR-214 antagonists have been reported to alleviate the phenotypic changes and proliferation of smooth muscle cells in vascular hyperproliferative diseases, including PAH [7]. In a consensus machine learning approach study, it was found that miR-636 and miR-187-5p could predict PAH diagnosis with high accuracy [8]. These studies suggest that miRNAs may be new therapeutic targets. Therefore, the identification of diagnostic biomarkers can identify PAH risk patients as soon as possible or provide new insights into the disease pathogenesis. Previous studies have found that miR-325 alleviates myocardial fibrosis after myocardial infarction by downregulating GLI1 [9]. However, there are few reports on miR-325's effect on right ventricular fibrosis with PAH. Therefore, this study wanted to explore miR-325-3p's role in right ventricular fibrosis and its related mechanisms in PAH.

Human epididymis protein 4 (HE4) has been reported to act as a fibroblast-derived mediator of renal fibrosis [10, 11]. Zhang et al. reported that hypoxia-induced HE4 promotes extracellular matrix accumulation and renal fibrosis [12]. Nagy Jr et al. found that HE4 level was correlated with the severity of cystic fibrosis lung disease [13]. These studies have revealed the role of HE4 in fibrosis. HE4 plasma level is related to heart failure severity and has prognostic value, which can improve the risk assessment of heart failure [14]. Our previous clinical studies have found that HE4 can be used as a predictor of clinical deterioration in idiopathic PAH and right heart failure patients [15]. However, the role of HE4 has not been evaluated in right ventricular fibrosis in PAH rats. Activation of the PI3K/AKT pathway was closely associated with cardiac fibrosis [16]. It was reported that HE4 can regulate Rab23 protein expression, and downregulation of Rab23 will significantly inhibit the PI3/AKT signaling pathway [17]. Therefore, we want to study whether HE4 plays a role in cell fibrosis through the PI3K/AKT pathway. Through bioinformatics analysis, we found that there were targeted binding sites between miR-325-3p and HE4, and it was speculated that HE4 was the downstream target gene of miR-325-3p. So we wanted to explore the role of miR-325-3p and HE4 in right ventricular fibrosis in PAH further.

Based on the above background, we investigated miR-325-3p/HE4's role in the occurrence and development of right ventricular fibrosis in PAH rats through the establishment of a PAH-induced right ventricular fibrosis model and in vivo and in vitro knockdown and overexpression vector experiments.

## 2. Materials and Methods

### 2.1. Animal Model

A total of 30 male SD rats with similar body weight were randomly divided into the Sham, PAH, and miR-325-3p group. Each group had 10 rats. The Sham group was intraperitoneally injected with normal saline. PAH group rats were treated as follows: 1% MCT solution was injected intraperitoneally at one time at 60 mg/kg [18] for 4 weeks, and then 1 × 107 TU LV-vector was injected into the tail vein. The treatment of the miR-325-3p group was as follows: 1% MCT solution was injected intraperitoneally at one time at 60 mg/kg [18] for 4 weeks, and then, 1 × 10^7^ TU LV-miR-325-3p was injected into the tail vein [9, 19]. Then, we detected the right ventricular systolic pressure (RVSP, mmHg) and right ventricular hypertrophy index (RVHI, %). All animal experiments have been approved by the ethics committee of Hunan Provincial People's Hospital (Approval number: 2019-06).

### 2.2. HE Staining

The rat pulmonary artery and right ventricle were sectioned in paraffin wax, respectively. The slices were baked in the microwave at 60°C for 2 h. Then, the slices were placed in xylene for 15 min, 3 times. Each stage was placed in gradient alcohol for 5 min. Then, they were soaked with distilled water for 5 min, stained with hematoxylin for 3 min, rinsed with distilled water, and returned blue with PBS. After eosin staining for 5 s, they were rinsed with distilled water and dehydrated with graded alcohol (95-100%) for 5 min per grade. The samples were removed and placed in xylene for 10 min, twice; sealed with neutral gum; and observed under a microscope (BA210T, Motic). The degree of pulmonary arterial medial wall thickness was evaluated according to the quantitative analysis method proposed by Yang et al. [20].

### 2.3. Masson Staining

Paraffin sections were made from the right ventricle of rats. The sections were dewaxed into water, the water on the sections was shaken off, and an appropriate amount of nuclear dye was added to cover the whole tissue, and the staining lasted for 3-5 min. Then, we rinsed the staining solution completely with tap water, soaked the sections with distilled water, and then soaked the sections with weak alkaline solvent such as PBS or ammonia for 5 to 10 minutes to make the nuclei return blue. We removed the water from the slices, added appropriate amount of pulp dye to cover the whole tissue, stained for 2 min, and rinsed the dye with rinse solution and colored separation solution for about 30 s. After adding an appropriate amount of redyeing solution to cover the whole tissue, they were stained for 6-8 min. The samples were blown dry and transparent, sealed with neutral gum, and observed under a microscope (BA210T, Motic).

### 2.4. Extraction and Identification of Primary Rat Fibroblasts

According to the previous studies [21–23], adult SD rats were taken and the heart tissue was removed. After digestion, the culture flask was placed in an incubator at 37°C and 5% CO_2_, and the adhesion was carried out for 50-60 min. Myocardial cell adhered to the wall more slowly than myocardial fibroblasts, so myocardial cell and myocardial fibroblasts could be separated. The culture flask was removed and gently shaken several times. The suspension containing the myocardial cell was transferred into a 50 mL centrifuge tube and centrifuged at 1000 rpm for 10 min. The supernatant was discarded, and the cells were resuspended in 10% FBS DMEM supplemented with BrdU. After 24 h, the inoculated myocardial cell was changed and washed twice with PBS to remove dead cells and unattached cells. More than 95% of the nonmyocardial cells obtained from primary culture were myocardial fibroblasts after 2-3 generations. Follow-up experiments on myocardial fibroblasts were performed 24 h after the cells reached the secondary fusion without serum starvation. Immunofluorescence (IF) detected Vimentin expression.

### 2.5. Cell Culture and Treatment

Rat primary myocardial fibroblasts were cultured in DMEM high glucose medium with 10% FBS. In order to explore miR-325-3p's effect on myocardial cell fibrosis, we constructed miR-325-3p mimic cell lines and grouped them into the control, model (Ang II), NC mimic, and miR-325-3p mimic groups. In the model group, myocardial fibroblasts were transfected with 1 *μ*M Ang II plasmid and incubated for 48 h to establish a fibrosis model. NC mimic treatment was treated as follows: on the basis of the model, myocardial fibroblasts were transfected with NC mimic interference sequences. The miR-325-3p mimic group was treated as follows: on the basis of the model, myocardial fibroblasts were transfected with miR-325-3p mimic plasmid. To explore HE4's effect on myocardial fibroblast fibrosis, si-HE4 and oe-HE4 cell lines were constructed and grouped as follows: the control, model (Ang II), si-NC, and si-HE4 groups and the control, model (Ang II), oe-NC, and oe-HE4 groups. The si-NC group was treated as follows: on the basis of the model, myocardial fibroblasts were transfected with si-NC interfering sequences. In the si-HE4 group, myocardial fibroblasts were transfected with si-HE4 plasmid on the basis of the model. In the oe-NC group, myocardial fibroblasts were transfected with the oe-NC interfering sequence on the basis of the model. The oe-NC group was treated as follows: myocardial fibroblasts were transfected with HE4 overexpressed plasmid on the basis of the model. In addition, we transfected miR-325-3p mimics and oe-HE4 simultaneously. The specific groups were as follows: the Ang II, NC mimic, miR-325-3p mimic, miR-325-3p mimic+oe-NC, and miR-325-3p mimic+oe-HE4 groups. Then, we added PI3K inhibitor LY294002 to study the effects of HE4 in cell fibrosis by the PI3K/AKT pathway. According to a previous study, 10 *μ*M PI3K inhibitor LY294002 (HY-10108, MedChemExpress) was added to pretreat cells for 30 min [24]. The specific groups were as follows: the Ang II, oe-NC, oe-HE4, and oe-HE4+LY294002 groups.

### 2.6. Quantitative Real-Time PCR (qRT-PCR)

Total RNA was extracted from cells and tissues by Trizol method, and cDNA was obtained by reverse transcription using the mRNA reverse transcription kit (CW2569, CWBIO, China). Primers for miR-325-3p, HE4, Acta2, collagen I, collagen III, MMP2, MMP9, U6, and GAPDH are shown in Table 1. The PCR reaction system was prepared by adding fluorescent dye. The DNA was amplified by a fluorescence quantitative PCR instrument (PIKOREAL96, Thermo, USA), and the fluorescence signal was monitored in real time to obtain the amplification curve and fusion curve of each gene. U6 and GAPDH served as an internal reference, and the 2^-*ΔΔ*Ct^ method was applied to calculate the levels of genes in each sample.

### 2.7. Western Blot

Total proteins were extracted from cells and tissues by RIPA lysate (#P0013B, Beyotime) according to the instructions. Protein quantization was performed for each group according to the BCA method. Loading buffer (#MB2479, Meilunbio) was mixed with SDS-PAGE. The mixture was heated in a boiling water bath at 100°C for 5 min, and the proteins were adsorbed on PVDF membrane by gel electrophoresis. The proteins were sealed with 5% skim milk solution for 90 min at room temperature and washed with PBST for 3 times, 5 min each time. The primary antibodies used in this study include HE4 (PA5-80227, 0.3 *μ*g/mL, Thermo Fisher), collagen I (66366-1-Ig, 1 : 2000, Proteintech), collagen III (22734-1-AP, 1 : 1000, Proteintech), MMP2 (22734-1-AP, 1 : 1000, Proteintech), MMP9 (27306-1-AP, 1 : 1000, Proteintech), PI3K (67071-1-Ig, 1 : 5000, Proteintech), p-PI3K (ab182651, 1 : 5000, Abcam), AKT (10176-2-AP, 1 : 1000, Proteintech), p-AKT (28731-1-AP, 1 : 3000, Proteintech), and GAPDH (10494-1-AP, 1 : 5000, Proteintech). Primary antibodies were incubated overnight at 4°C. Then, secondary antibodies were incubated for 90 min. ECL was color exposed, and protein bands were detected by the Odyssey Infrared Imaging System (Li-Cor Biosciences, Lincoln, NE, USA), and GAPDH served as the internal reference to detect the expression level.

### 2.8. IF

The Vimentin and *α*-SMA expressions in myocardial fibroblasts were detected by IF. The slice was removed and cleaned with PBS for 2-3 times. The slice was fixed with 4% paraformaldehyde for 30 min and rinsed with PBS for 3 times, 5 minutes each time. Then, 0.5% Triton X-100 was used to permeate for 30 min at 37°C. After rinsing with PBS, 5% BSA was sealed at 37°C for 1 h, Vimentin and *α*-SMA primary antibodies were incubated at 4°C overnight, and PBS was rinsed 3 times for 5 min each time. Then, secondary antibodies were incubated at 37°C for 90 min. DAPI was stained at 37°C for 10 min. The tablet was sealed and observed under a fluorescence microscope.

### 2.9. Bioinformatics Prediction and Dual-Luciferase Reporter Assay

Starbase predicted miR-325-3p and HE4 binding sites. Then, we constructed wild-type (WT) or mutant (MUT) miR-325-3p fragments and inserted them into the pmirGLO vector (Promega). According to the instructions, Lipofectamine 3000 reagent (Thermo Fisher Scientific, USA) was applied to transfect the recombinant vector into the cells; mimic NC and miR-325-3p mimics were simultaneously transferred into the cells. Finally, the luciferase activity was determined by Nano-Glo (Promega).

### 2.10. Statistical Analysis

The data of this study were statistically analyzed by GraphPad Prism 8.0 statistical software. The data were expressed as mean ± standard deviation (mean ± SD) and repeated at least 3 times. Student's *t-*test was utilized between two groups, and one-way analysis of variance (ANOVA) was applied for comparison between multiple groups. Pearson correlation coefficient analyzed miR-325-3p and HE4, collagen I, collagen III, MMP2, and MMP9 correlation. *P* < 0.05 considered the difference to be statistically significant.

## 3. Results

### 3.1. Overexpression of miR-325-3p Alleviated Myocardial Fibrosis in Rats with PAH

**Figure 1:**
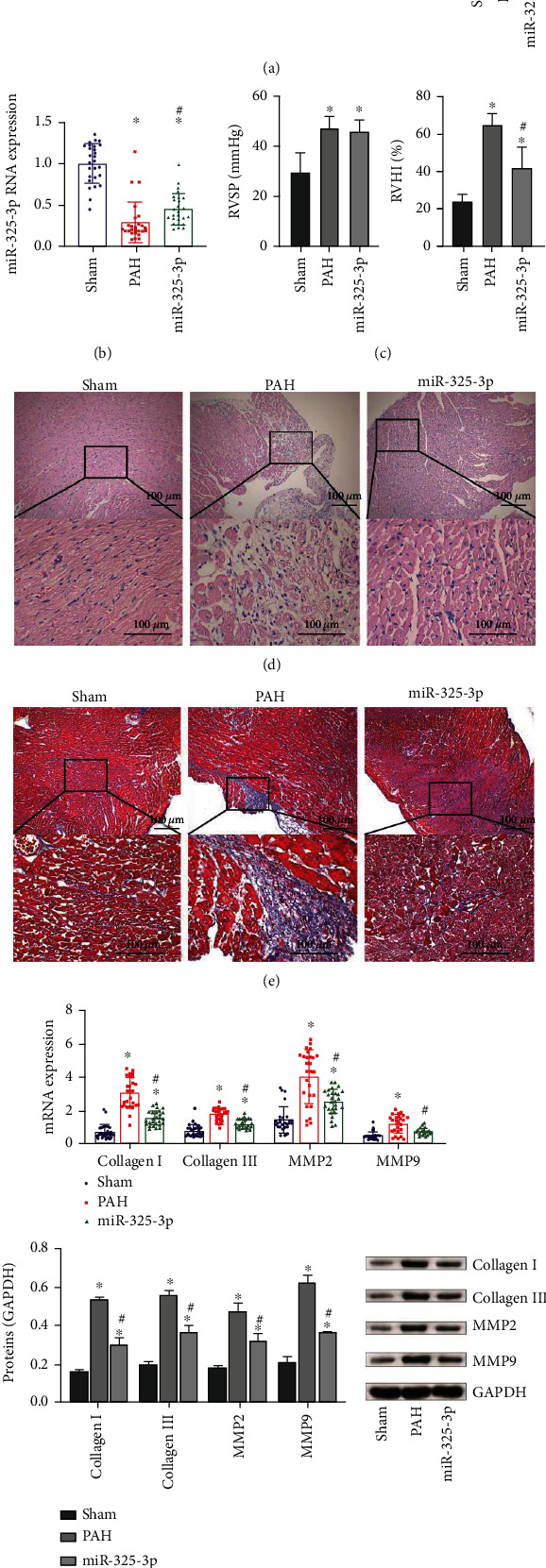
Overexpression of miR-325-3p alleviated myocardial fibrosis in rats with PAH. (a) HE staining of the pulmonary artery and quantitative statistics of pulmonary arterial medial wall thickness (%). (b) miR-325-3p expression in myocardial tissue was measured by qRT-PCR. (c) RVSP and RVHI of rats. (d) HE staining of the right ventricle (×100, 100 *μ*m). (e) Masson staining of the right ventricle (×100, 100 *μ*m). (f, g) qRT-PCR and Western blot were performed to measure collagen I, collagen III, MMP2, and MMP9 expressions in heart tissue. ^∗^*P* < 0.05 vs. Sham; ^#^*P* < 0.05 vs. PAH.

We first explored miR-325-3p's effect on myocardial fibrosis in PAH rats. As shown in Figure 1(a), HE staining results of the pulmonary artery showed that the distance between the pulmonary arterioles (fibrous layer) and intima (endocortex) was significantly widened in the PAH group, the thickness of the medial layer (smooth muscle layer) was increased, and the proportion of the medial area to the total cross-sectional area of pulmonary arterioles was increased. This indicated that the PAH model was established successfully. There was no significant difference in pulmonary arterial medial wall thickness between the miR-325-3p group and PAH group (Figure 1(a)). Compared with the Sham group, miR-325-3p expression in the PAH group was decreased, while miR-325-3p expression was increased in the miR-325-3p group (Figure 1(b)). Compared with the Sham group, RVSP increased in the PAH group. There was no significant difference in RVSP between the PAH group and miR-325-3p group. Compared with the Sham group, RVHI increased in the PAH group, while RVHI decreased in the miR-325-3p group (Figure 1(c)). HE staining of the right ventricle of rats showed that, compared with the Sham group, the PAH group showed atrophy of muscle cells, more inflammatory cells, obvious inflammation, and disarray of myocardial structure, while symptoms were alleviated in the miR-325-3p group (Figure 1(d)). Masson staining showed that the PAH group showed significant fibrosis compared with the Sham group, and fibrosis was alleviated in the miR-325-3p group (Figure 1(e)). Then, we detected collagen I, collagen III, MMP2, and MMP9 levels. Collagen I and collagen III expressions were also increased in the PAH group. In addition, MMP2 and MMP9 expressions were also increased. Compared with the PAH group, collagen I and collagen III expressions in the miR-325-3p group were decreased. In addition, MMP2 and MMP9 expressions also decreased (Figure 1(f)). These results suggested that PAH induced myocardial fibrosis in rats, and miR-325-3p overexpression alleviated myocardial fibrosis in rats with PAH.

### 3.2. HE4 Was Negatively Correlated with miR-325-3p Expression

**Figure 2:**
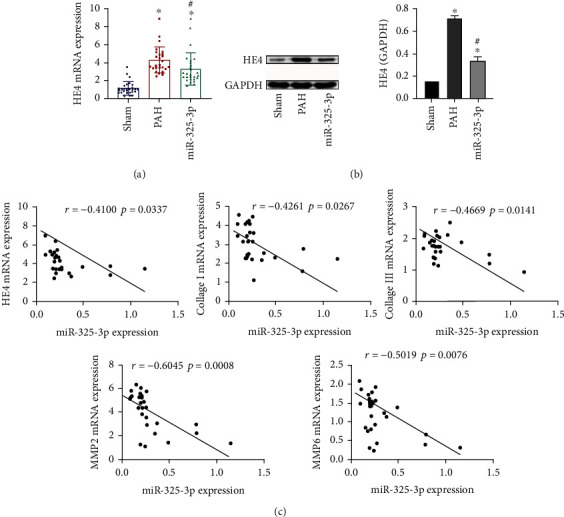
HE4 was negatively correlated with miR-325-3p expression. (a) HE4 expression in right heart tissue was assessed by qRT-PCR. (b) Western blot was used to detect the expression of HE4 in right heart tissue. (c) Pearson correlation coefficient was used to analyze miR-325-3p and HE4, collagen I, collagen III, MMP2, and MMP9 correlation. ^∗^*P* < 0.05 vs. Sham; ^#^*P* < 0.05 vs. PAH.

Next, we examined the expression of HE4 in heart tissue. As shown in Figures 2(a) and 2(b), compared with the Sham group, HE4 expression in the PAH group was increased, while HE4 expression in the miR-325-3p group was slightly decreased. Pearson correlation coefficient analysis showed that miR-325-3p was negatively correlated with HE4, collagen I, collagen III, MMP2, and MMP9 (Figure 2(c)). The above results showed that HE4 was negatively correlated with miR-325-3p expression.

### 3.3. miR-325-3p-Targeted Regulation of HE4 to Inhibit Myocardial Fibroblast Fibrosis

**Figure 3:**
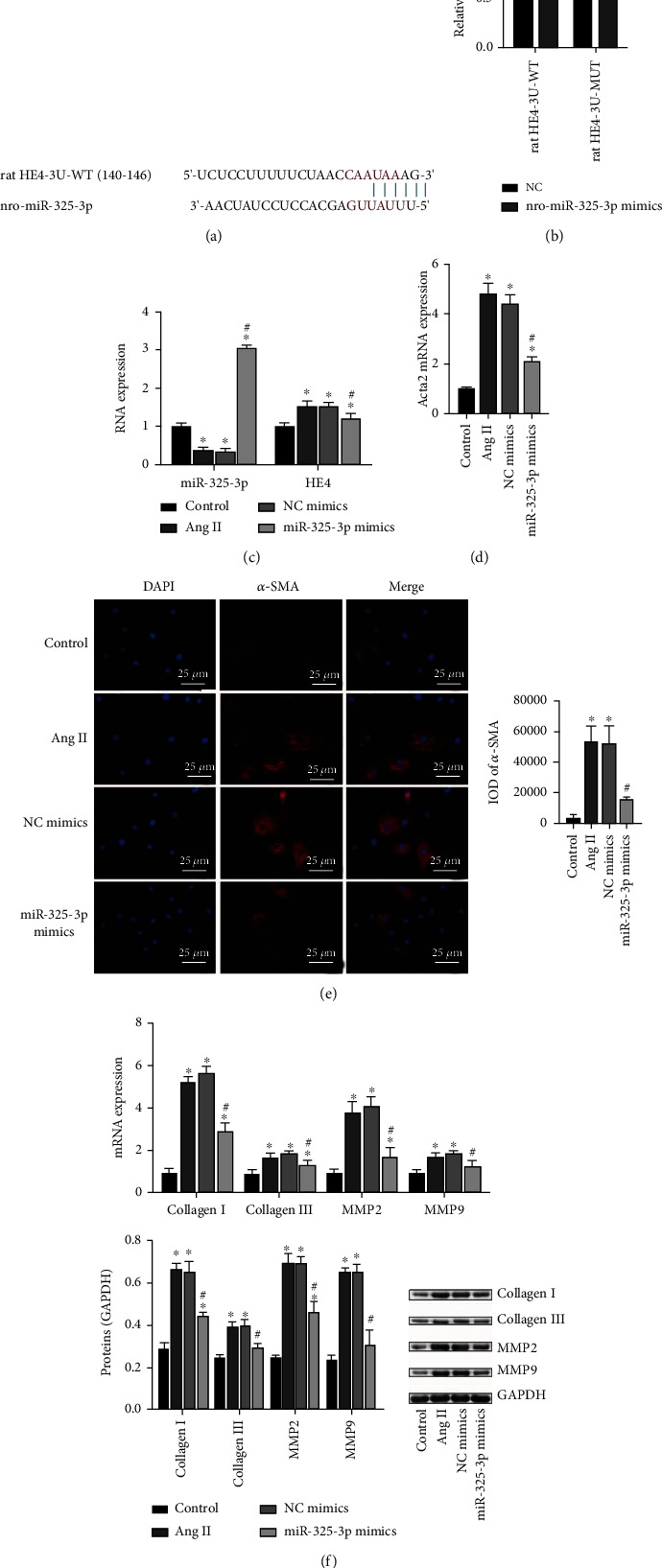
miR-325-3p-targeted regulation of HE4 to inhibit myocardial fibroblast fibrosis. (a) Starbase was applied to predict miR-325-3p and HE4 binding sites. (b) Dual-luciferase reporter assay experiments proved that miR-325-3p targeted HE4. (c) miR-325-3p and HE4 expressions were assessed by qRT-PCR. (d) Expression of Acta2 was measured by qRT-PCR. (e) *α*-SMA expression was detected by IF (×400, 25 *μ*m). (f) qRT-PCR and Western blot were utilized to detect collagen I, collagen III, MMP2, and MMP9 levels. ^∗^*P* < 0.05 vs. control; ^#^*P* < 0.05 vs. NC mimics.

Studies have found that miR-325 alleviated myocardial fibrosis after myocardial infarction by downregulating GLI1 [9]. We predicted that miR-325-3p had a binding site with HE4 through Starbase website (Figure 3(a)). Dual-luciferase reporter assay demonstrated that miR-325-3p targeted HE4 (Figure 3(b)). qRT-PCR results showed that compared with the control group, miR-325-3p expression in the model group was decreased, and miR-325-3p expression was more significantly increased after transfecting miR-325-3p mimics (Figure 3(c)). This indicated that miR-325-3p mimic transfecting was successful. Compared with the control group, HE4 expression in the model group was increased, and after transfecting miR-325-3p mimics, HE4 expression was decreased (Figure 3(c)). Then, we detected fibrosis indicators. qRT-PCR results showed that, compared with the control group, Acta2 expression in the model group was increased, and Acta2 expression was decreased after transfecting miR-325-3p mimics (Figure 3(d)). IF results also showed that *α*-SMA expression was increased in the model group compared with the control group and decreased after transfecting miR-325-3p mimics (Figure 3(e)). qRT-PCR and Western blot results also showed that compared with the control group, collagen I and collagen III expressions in the model group were increased. After transfecting miR-325-3p mimics, collagen I and collagen III expressions were decreased. In addition, compared with the control group, MMP2 and MMP9 expressions in the model group were increased. After transfecting miR-325-3p mimics, MMP2 and MMP9 expressions decreased (Figure 3(f)). All in all, miR-325-3p-targeted regulation of HE4 inhibited myocardial fibroblast fibrosis.

### 3.4. Knockdown of HE4 Inhibited Myocardial Fibroblast Fibrosis

**Figure 4:**
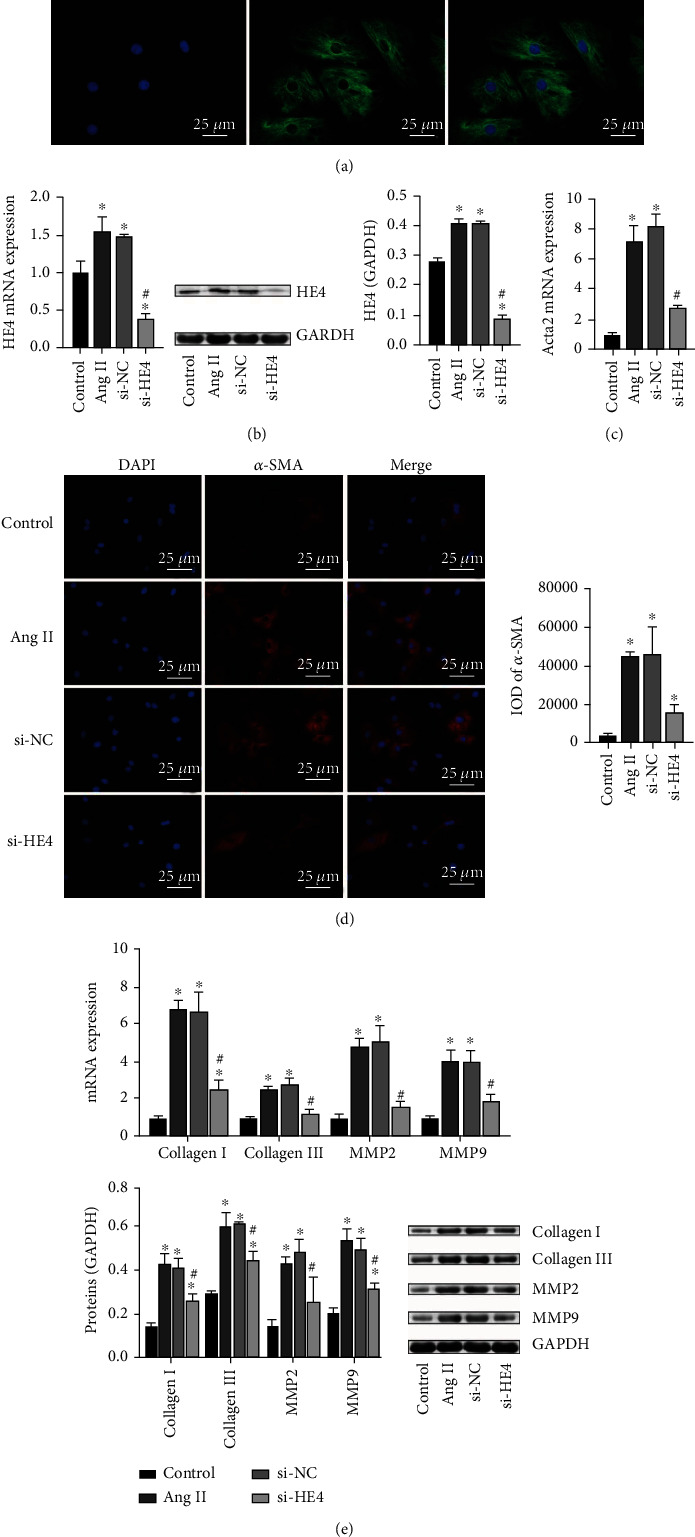
Knockdown of HE4 inhibited myocardial fibroblast fibrosis. (a) Identification of primary rat myocardial fibroblast (×400, 25 *μ*m). (b) Expression of HE4 was tested by qRT-PCR and Western blot. (c) Acta2 expression was tested by qRT-PCR. (d) IF determined *α*-SMA expression (×400, 25 *μ*m). (e) qRT-PCR and Western blot were utilized to detect collagen I, collagen III, MMP2, and MMP9 levels. ^∗^*P* < 0.05 vs. control; ^#^*P* < 0.05 vs. si-NC.

Then, we extracted the rat primary myocardial fibroblast. The results of IF staining Vimentin showed that the primary rat myocardial fibroblasts were isolated successfully (Figure 4(a)). To study HE4's effect on PAH myocardial fibroblast fibrosis, we constructed a cell fibrosis model and transfected si-HE4 plasmid. As shown in Figure 4(b), compared with the control group, HE4 expression in the model group was increased, and HE4 expression was decreased after transfecting si-HE4. This indicated that the transfection of si-HE4 was successful. Then, we detected fibrosis indicators. qRT-PCR results revealed that compared with the control group, Acta2 expression in the model group was increased, and Acta2 expression was decreased after transfecting si-HE4 (Figure 4((c))). The IF results also revealed that *α*-SMA expression in the model group was increased compared with the control group, and *α*-SMA expression was decreased after transfecting si-HE4 (Figure 4(d)). qRT-PCR and Western blot results also revealed that compared with the control group, collagen I and collagen III expressions in the model group were increased. After transfecting si-HE4, collagen I and collagen III expressions were decreased. In addition, compared with the control group, MMP2 and MMP9 expressions in the model group were increased. After transfecting with si-HE4, MMP2 and MMP9 expressions decreased (Figure 4(e)). Therefore, knockdown of HE4 inhibited myocardial fibroblast fibrosis.

### 3.5. HE4 Promoted Myocardial Fibroblast Fibrosis and Activated the PI3K/AKT Pathway

**Figure 5:**
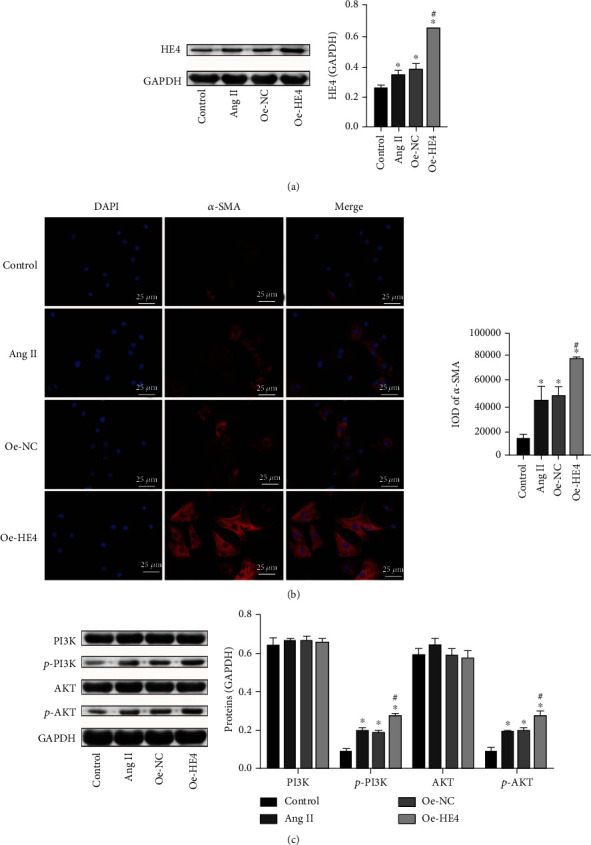
HE4 promoted myocardial fibroblast fibrosis and activated the PI3K/AKT pathway. (a) Western blot was applied to test HE4 expression. (b) *α*-SMA expression was detected by IF (×400, 25 *μ*m). (c) Western blot was used to examine PI3K, p-PI3K, ATK, and p-AKT levels. ^∗^*P* < 0.05 vs. control; ^#^*P* < 0.05 vs. oe-NC.

Activation of the PI3K/AKT pathway is closely related to cardiac fibrosis [16]. It has been reported that the combination of HDAC3 and HE4 activated the PI3K/AKT pathway in ovarian cancer [25]. It has been reported that the combination of HDAC3 and HE4 activates the PI3K/AKT pathway in ovarian cancer [18]. To investigate HE4 and PI3K/AKT pathway's effect on PAH myocardial fibroblast fibrosis, we constructed a cell fibrosis model and transfected oe-HE4 plasmid. As shown in Figure 5(a), compared with the control group, HE4 expression in the model group was increased, and HE4 expression increased more significantly after oe-HE4 transfection. This indicated successful transfection of oe-HE4. Then, we measured fibrosis indicators. IF results also showed that compared with the control group, *α*-SMA expression in the model group was increased, and *α*-SMA expression was more significantly increased after oe-HE4 transfection (Figure 5(b)). Western blot results showed that p-PI3K and p-AKT expressions in the model group were increased compared with the control group. After transfecting with oe-HE4, p-PI3K and p-AKT expressions increased significantly (Figure 5(c)). These results indicated that HE4 promoted myocardial fibroblast fibrosis by activating the PI3K/AKT pathway.

### 3.6. Overexpression of HE4 Could Reverse the Effect of miR-325-3p Mimics

**Figure 6:**
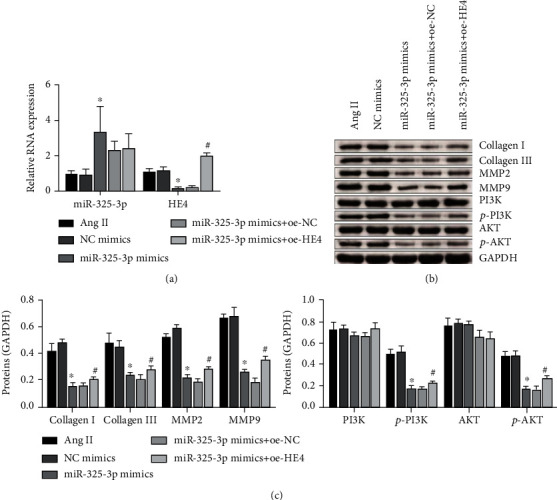
Overexpression of HE4 could reverse the effect of miR-325-3p mimics. (a) qRT-PCR was performed to detect the expression of miR-325-3p and HE4. ^∗^*P* < 0.05 vs. NC mimics; ^#^*P* < 0.05 vs. miR-325-3p mimics+oe-NC. (b) Western blot was used to measure collagen I, collagen III, MMP2, MMP9, PI3K, p-PI3K, AKT, and p-AKT levels. (c) Quantitative statistics of (b) proteins. ^∗^*P* < 0.05 vs. NC mimics; ^#^*P* < 0.05 vs. miR-325-3p mimics+oe-NC.

As shown in Figure 6(a), levels of miR-325-3p and HE4 were detected by qRT-PCR. The results showed that compared with the NC mimic group, the expression level of miR-325-3p in the miR-325-3p mimic group was increased, indicating that the transfection of miR-325-3p mimics was successful. Compared with the miR-325-3p mimic+oe-NC group, the expression level of HE4 in the miR-325-3p mimics+oe-HE4 group was increased, indicating that the transfection of oe-HE4 was successful. Then, Western blot examined the expressions of collagen I, collagen III, MMP2, MMP9, PI3K, p-PI3K, AKT, and p-AKT. Compared with the NC mimic group, collagen I, collagen III, MMP2, MMP9, p-PI3K, and p-AKT levels in the miR-325-3p mimic group were decreased, while PI3K and AKT levels were not changed. Collagen I, collagen III, MMP2, MMP9, p-PI3K, and p-AKT levels increased in the miR-325-3p mimic+oe-HE4 group compared with the miR-325-3p mimic+oe-NC group. The expression levels of PI3K and AKT remained unchanged (Figures 6(b) and 6(c)). These results suggested that overexpression of HE4 could reverse the effect of miR-325-3p mimics.

### 3.7. HE4 Affected Myocardial Fibroblast Fibrosis through the PI3K/AKT Pathway

**Figure 7:**
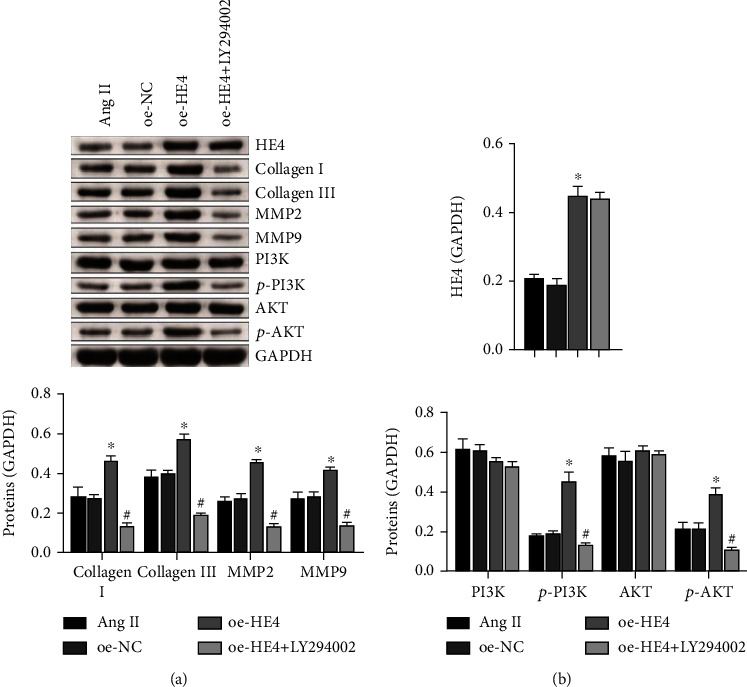
HE4 affected myocardial fibroblast fibrosis through the PI3K/AKT pathway. (a) The expressions of HE4, collagen I, collagen III, MMP2, MMP9, PI3K, p-PI3K, AKT, and p-AKT were detected by Western blot. (b) Quantitative statistics of (a) proteins. ^∗^*P* < 0.05 vs. oe-NC; ^#^*P* < 0.05 vs. oe-HE4.

We then added PI3K inhibitor LY294002 to study the effects of HE4 in cell fibrosis by the PI3K/AKT pathway. As shown in Figures 7(a) and 7(b), the expression level of HE4 in the oe-HE4 group was increased compared with that in the oe-NC group. Collagen I, Collagen III, MMP2, MMP9, p-PI3K, and p-AKT expression levels also increased, while PI3K and AKT expression levels remained unchanged. After the addition of inhibitor LY294002, the expression level of HE4 in the oe-HE4+LY294002 group was basically unchanged compared with that in the oe-HE4 group. Collagen I, collagen III, MMP2, MMP9, p-PI3K, and p-AKT decreased, while the expression levels of PI3K and AKT remained unchanged. These results showed that HE4 affected myocardial fibroblast fibrosis through the PI3K/AKT pathway.

## 4. Discussion

PAH is a fatal cardiovascular disease with cancer-like phenotype [26]. However, treatment for PAH is limited. In our paper, we investigated miR-325-3p and HE4 effects on right ventricular fibrosis in PAH rats by constructing a right ventricular fibrosis model and myocardial fibroblast fibrosis model. Our study suggests miR-325-3p may alleviate right ventricular fibrosis in rats with PAH by targeting HE4 to regulate the PI3K/AKT signaling pathway. This is the first report on miR-325-3p-targeting HE4 mechanism in right ventricular fibrosis in rats with PAH.

Right ventricular myocardial fibrosis in hypoxic PAH has been reported to be associated with local renin-angiotensin system activation [27]. Excessive adipose tissue is the cause of the deterioration of circulating Ang II, which leads to the occurrence of cardiac fibrosis [28]. In severe right ventricular dysfunction, both myofibril- and fibrosis-mediated stiffness increased right ventricular myocardial stiffness [29]. Studies have reported that overexpression of miR-150 has a protective effect on hypoxia-induced myocardial fibrosis [30]. Our study was consistent with previous reports. We found that collagen I and collagen III expressions were increased in the right ventricular in the PAH group. In the miR-325 group, collagen I and collagen III expressions were decreased. Inhibition of MMPs could attenuate the monocrotaline-induced right ventricular remodeling [31]. Sun et al. reported that miR-325-3p inhibited renal inflammation and fibrosis in both DN cell and mouse models by targeting CCL19 [32]. We found that compared with the sham group, MMP2 and MMP9 expressions were increased in the PAH group. MMP2 and MMP9 expressions in the miR-325-3p group were also decreased. This suggests that miR-325-3p overexpression alleviates myocardial fibrosis in rats with PAH.

It was reported that miR-325-3p protected the mouse heart after myocardial infarction through inhibiting RIPK3 and programmed necrosis [33]. LncRNA MEG3 regulates the expression of TRPV4 by phagocytosis of miR-325-3p in sponge, thereby increasing hypoxia-induced myocardial cell injury in rats [34]. This suggests that miR-325 is involved in right ventricular fibrosis in PAH development. HE4 is a secreted protein expressed in activated fibroblasts that can aggravate tissue fibrosis. As a secretory factor, HE4 can activate myocardial fibroblasts and thus induce myocardial interstitial fibrosis [35]. HE4 may be a promising biomarker for the evaluation of persistent fibrosis. Our study showed that HE4 was negatively correlated with miR-325-3p expression, and miR-325-3p targeted HE4. After transfecting miR-325-3p mimics and si-HE4, collagen I and collagen III expressions were decreased. In addition, MMP2 and MMP9 expressions were also decreased. This indicated that miR-325-3p-targeted regulation of HE4 inhibited myocardial cell fibrosis and HE4 knockdown inhibited myocardial cell fibrosis.

Activation of the PI3K/AKT pathway was closely associated with cardiac fibrosis [16]. It has been reported that inhibiting miR-1 may attenuate right ventricular fibrosis in PAH model rats, a mechanism that may involve the PI3K/AKT signaling pathway [36]. In addition, recent reports indicate that YWHAE, as a HE4 interacting protein, can affect ovarian cancer malignant behavior through regulating PI3K/AKT and MAPK pathways [37]. In this study, after transfection with oe-HE4, PI3K/AKT pathway-related protein p-PI3K and p-AKT expressions were significantly increased, and *α*-SMA expression was increased. This suggested that HE4 promoted myocardial fibroblast fibrosis and activated the PI3K/AKT pathway. After adding PI3K inhibitor LY294002, we could conclude that HE4 affected myocardial fibroblast fibrosis through the PI3K/AKT pathway. However, there are some limitations to our article. Our research is not deep enough, and we did not test the expression level of Ang II in PAH rats. In the future, we will detect the expression level of Ang II in PAH rats and further study the role of HE4 in right ventricular fibrosis in rats with PAH.

## 5. Conclusions

Our findings suggest that miR-325 may alleviate right ventricular fibrosis in rats with PAH by targeting HE4 to regulate PI3K/AKT signaling. Our research provides a theoretical basis for right ventricular fibrosis in PAH pathogenesis and also provides a new therapeutic idea for the treatment of right ventricular fibrosis in PAH.

## Figures and Tables

**Table 1:** The primers used in this study.

Name	5′-3′
miR-325-3p-F	TTTATTGAGCACCTCCTATCAAA
miR-325-3p-RT	GCTGTCAACGATACGCTACGTAAC
HE4-F	AGAGGTTGCTTCTGCTGGAC
HE4-R	TGTGGGACCAGGACGAAATG
MMP9-F	CCCCGAGACCTGAAAACCTCCAAC
MMP9-R	GGCCTTTAGTGTCTCGCTGTCCA
MMP2-F	GGTGGCAATGGAGATGGACA
MMP2-R	CCGGTCATAATCCTCGGTGG
Collagen I-F	CTGGCTCTCCTGGTACCCCT
Collagen I-R	GGACCACGTTCACCACTTGCT
Collagen III-F	CCCCTCTCTTATTTTGGCACAG
Collagen III-R	CGCAGACACATATTTGACATGG
Acta2-F	GTCAGGAATCCCGTGAAGCA
Acta2-R	CATTGTCACACACAAGGGCG
GAPDH-F	ACAGCAACAGGGTGGTGGAC
GAPDH-R	TTTGAGGGTGCAGCGAACTT
U6-F	GCCTACAGCCATACCACCCGGAA
U6-R	CCTACAGCACCCGGTATCCCA

## Data Availability

The data used to support the findings of this study are available from the corresponding author upon request.
